# Roles and regulatory mechanisms of autophagy in the pathology of spinal cord injury

**DOI:** 10.1093/burnst/tkag058

**Published:** 2026-08-29

**Authors:** Zhiwan Chen, Jiajun Deng, Ruijin Huang, Zhisheng Ji

**Affiliations:** Department of Orthopedics, The First Affiliated Hospital, Jinan University, Guangzhou, Guangdong, 510632, P. R. China; Department of Orthopedics, The First Affiliated Hospital, Jinan University, Guangzhou, Guangdong, 510632, P. R. China; Institute of Neuroanatomy, University of Bonn and University Hospital of Bonn, Germany; Department of Orthopedics, The First Affiliated Hospital, Jinan University, Guangzhou, Guangdong, 510632, P. R. China

**Keywords:** spinal cord injury, autophagy, inflammation, oxidative stress, axonal regeneration, autophagic flux, autophagy signaling pathways, neuroprotection, autophagymodulating drugs

## Abstract

Spinal cord injury (SCI) remains a major clinical challenge due to its limited therapeutic options and the resulting severe functional impairments. Autophagy is an essential intracellular degradation process that plays a critical role in maintaining cellular homeostasis by degrading and recycling abnormal proteins and toxic substances. Recent studies have shown that autophagy significantly regulates several key pathological processes in the acute phase of SCI, including oxidative stress and neuroinflammation, and may also influence axonal regeneration during subsequent repair stages. Pharmacological modulation of autophagic flux has shown therapeutic promise and may exert neuroprotective effects. However, the precise relationship between autophagy activation or inhibition and functional recovery remains controversial and has not been fully elucidated. This review systematically summarizes recent advances in understanding the role and regulatory mechanisms of autophagy in the pathological processes of SCI, with particular emphasis on current controversies and unresolved issues. It also outlines potential pharmacological strategies targeting autophagy and proposes future research directions to support the clinical translation of these therapeutic approaches.

## Highlights

- Autophagic flux should be evaluated rather than inferred from static autophagy markers alone.
- Autophagy has stage- and cell-type-specific effects after spinal cord injury.
- Lysosomal function and mitophagy influence oxidative stress and neural repair after spinal cord injury.
- Treatment strategies targeting autophagy should consider both flux status and intervention timing.

## Background

Spinal cord injury (SCI) is a devastating neurological condition characterized by extensive and often irreversible damage to the spinal cord, leading to severe sensory, motor, and autonomic dysfunction [1]. With its increasing incidence worldwide, SCI has become a significant public health issue, imposing substantial physical, psychological, and socioeconomic burdens on individuals and healthcare systems [2]. Despite decades of research, effective clinical therapies remain limited, underscoring the need to better understand the complex pathological mechanisms underlying SCI and to develop novel therapeutic approaches.

**Figure 1:**
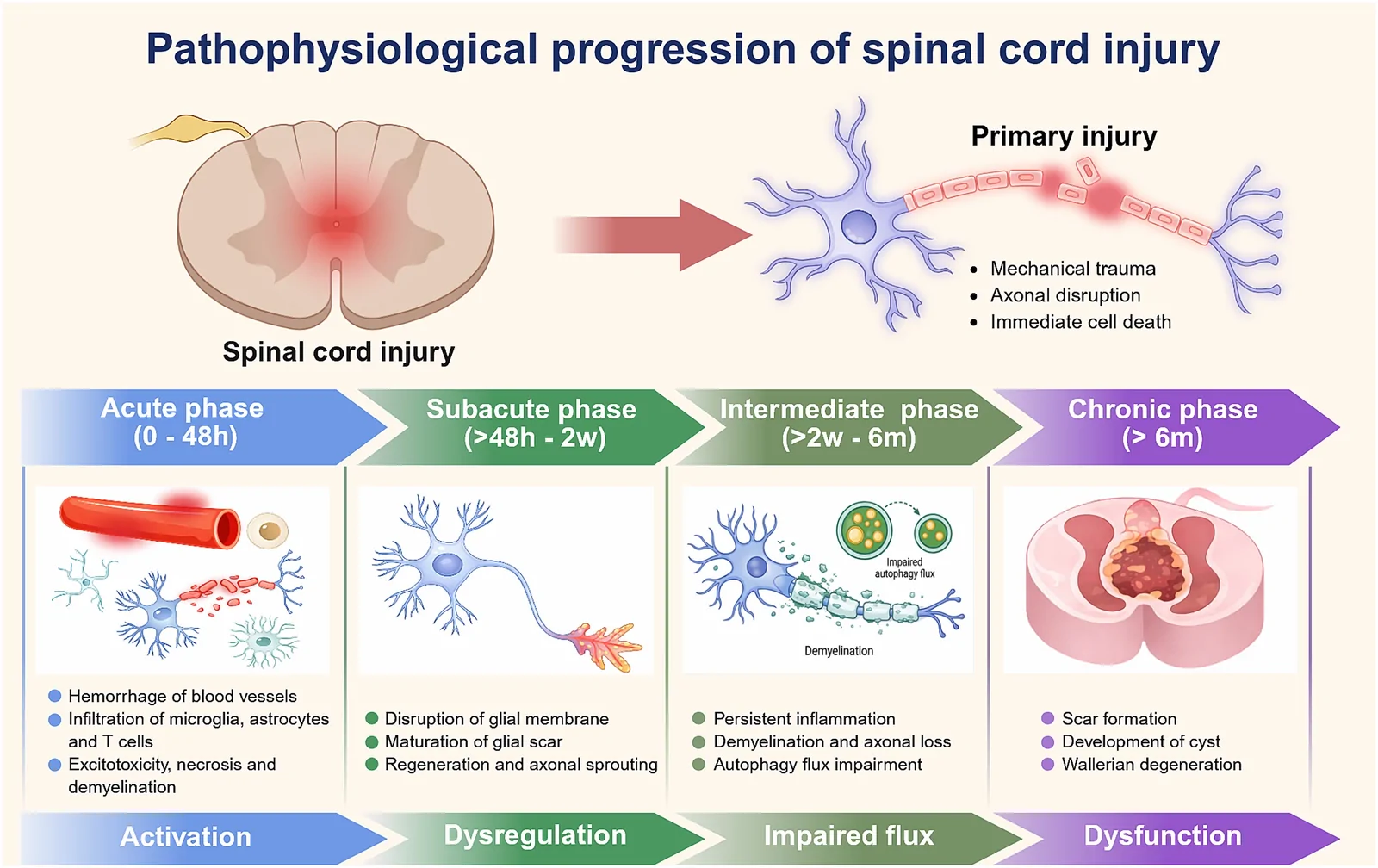
Pathophysiological mechanisms of SCI. SCI is classified into primary and secondary injuries. Secondary SCI is further divided into acute phase (0–48 hours), subacute phase (>48 hours–2 weeks), intermediate phase (>2 weeks—6 months), and chronic phase (>6 months). *SCI* spinal cord injury

From a pathophysiological standpoint, SCI is traditionally classified into primary and secondary injuries [3]. The primary injury results directly from initial mechanical trauma, causing immediate structural disruptions such as neuronal death, axonal rupture, spinal cord ischemia, hemorrhage, and acute inflammation. This initial damage is largely irreversible. However, subsequent secondary injuries—marked by progressive cellular and molecular events—often exacerbate tissue damage, leading to further neurological deterioration. Secondary injury progression is commonly divided into four overlapping temporal phases based on time and pathological characteristics: acute (0–48 hours post-injury), subacute (>48 hours–2 weeks), intermediate (>2 weeks–6 months), and chronic (>6 months) [4, 5] (Figure 1). Although the exact temporal boundaries may vary somewhat across studies, this framework provides a more complete description of SCI progression and helps better contextualize phase-dependent pathological events and therapeutic responses. The acute phase is particularly critical, as it sets the stage for subsequent pathological changes and significantly affects the eventual prognosis of SCI patients. Early pathological processes during the acute phase include vascular disruption, electrolyte imbalance, excitotoxicity from neurotransmitter release, oxidative stress, inflammatory cell infiltration, extensive edema formation, and necrosis [6].

Autophagy, a fundamental catabolic pathway first described by Belgian biochemist Christian de Duve in the 1960s, has emerged as a crucial cellular process involved in maintaining homeostasis under physiological and pathological conditions [7]. It functions by delivering damaged organelles, abnormal proteins, and other cytoplasmic constituents into lysosomes for degradation and recycling [8]. Under normal conditions, autophagy sustains cellular integrity by eliminating toxic substances and damaged components, thereby preventing disease onset. Conversely, in stressful environments—such as nutrient deprivation, hypoxia, or injury—autophagy serves as an adaptive response providing energy substrates and metabolic precursors. However, dysregulated or excessive autophagy can result in detrimental outcomes, including autophagic cell death or exacerbation of tissue injury [9]. This dual role has been increasingly recognized in various diseases, notably neurodegenerative disorders [10], cancer [11], ischemic injury, and trauma [12].

In recent years, significant attention has been directed toward exploring the role of autophagy in the pathogenesis and therapeutic modulation of SCI, particularly during the acute injury phase. Studies suggest that autophagy is intricately involved in critical processes during the acute phase, including neuronal survival, regulation of inflammation, and responses to oxidative stress, and may also influence axonal regeneration during subsequent repair stages. Enhancing autophagic flux has shown promise as a neuroprotective strategy in several preclinical SCI models, potentially reducing apoptosis, mitigating inflammatory cascades, and alleviating oxidative damage. However, paradoxically, other studies have indicated that excessive autophagy activation under certain conditions may exacerbate neuronal loss and hinder functional recovery, underscoring the complexity and context-dependence of autophagic regulation in SCI pathology.

Despite recent advances, the precise regulatory mechanisms of autophagy during the acute phase of SCI, the optimal therapeutic time window, and the variations in autophagic responses across different injury severities and experimental models remain incompletely understood and are still subject to considerable debate. In addition, the translation of autophagy-targeted therapeutic strategies from basic research to clinical application faces substantial challenges, including the bidirectional effects of autophagy regulation, difficulties in achieving target specificity, and the need for a deeper understanding of its roles across different injury stages, including axonal regeneration during the subsequent repair phase.

Therefore, this review will systematically summarize recent advances in understanding the regulatory mechanisms and functional roles of autophagy in the pathological processes of SCI, critically analyze existing controversies and gaps in knowledge, and explore potential autophagy-targeted therapeutic strategies and future research directions, with the aim of providing new insights for clinical treatment of SCI.

## Review

### Autophagy and autophagic flux

Recent advances have shifted the focus of SCI research from simply determining whether autophagy is activated after injury to understanding whether autophagic flux is successfully completed and whether lysosomal function is preserved or restored. At the same time, autophagy is increasingly recognized as a dynamic regulator of secondary injury, extending beyond intracellular degradation to influence neuroinflammation, microglial responses, and tissue repair. Against this background, understanding the fundamental process of autophagy and autophagic flux provides the basis for interpreting their stage-dependent roles in SCI [13–17].

Autophagy is a highly conserved intracellular degradation and recycling process that plays a critical role in maintaining cellular homeostasis and responding to stress conditions. This process is conserved across eukaryotic cells, functioning through the degradation and recycling of damaged organelles, misfolded proteins, invading pathogens, and other abnormal intracellular components, thereby supporting cellular homeostasis and renewal. Autophagy is classified into three distinct types: macroautophagy, microautophagy, and chaperone-mediated autophagy (CMA) [18].

Macroautophagy, the most extensively studied form and commonly referred to simply as “autophagy”, involves the formation of double-membrane vesicles known as autophagosomes. These autophagosomes encapsulate intracellular substrates and ultimately fuse with lysosomes to form autolysosomes, where degradation takes place. In contrast, microautophagy directly sequesters cytoplasmic components through invaginations of the lysosomal membrane, facilitating rapid and less selective degradation [19]. CMA selectively targets specific cytosolic proteins containing distinct amino acid sequences, utilizing chaperone proteins such as Hsc70 to facilitate their transport across lysosomal membranes for degradation [18]. In addition to these broad categories, autophagy can also be classified according to substrate selectivity. Selective autophagy refers to the targeted degradation of specific intracellular cargoes through cargo receptors or adaptor proteins that link these substrates to LC3-positive autophagosomal membranes. Major forms of selective autophagy include mitophagy, reticulophagy, pexophagy, lipophagy, aggrephagy, and xenophagy [20]. Among them, mitophagy is particularly relevant to SCI, as mitochondrial dysfunction, reactive oxygen species (ROS) accumulation, and disrupted energy metabolism are central features of secondary injury [21]. Therefore, mitophagy should be considered a specialized form of selective macroautophagy rather than a process parallel to autophagy [22]. This distinction provides an important conceptual basis for the later discussion of oxidative stress, mitochondrial quality control, and autophagy-mediated neuroprotection after SCI.

Importantly, recent SCI-related evidence suggests that the field is beginning to expand beyond canonical macroautophagy [23]. A 2025 study reported that LAMP2A-mediated CMA modulates microglial polarization and inflammation in ischemia-induced SCI, indicating that selective autophagy pathways may also contribute to SCI pathology and repair [24].

Macroautophagy comprises several key stages: initiation, expansion and maturation of autophagosomes, fusion with lysosomes, and substrate degradation and recycling [25]. The initiation stage involves the formation of phagophores—cup-shaped isolation membranes derived from various intracellular membrane sources such as the endoplasmic reticulum (ER), Golgi apparatus, mitochondria, and endosomes. Phagophores subsequently elongate to engulf damaged organelles and other harmful cytoplasmic contents, eventually maturing into double-membrane autophagosomes. These mature autophagosomes are transported along intracellular microtubule networks and fuse with lysosomes, forming autolysosomes where enclosed substrates are degraded into basic molecules such as amino acids and fatty acids, which are then recycled back into the cytoplasm. This entire process, from autophagosome formation to substrate degradation and recycling, is termed autophagic flux [26].

**Figure 2:**
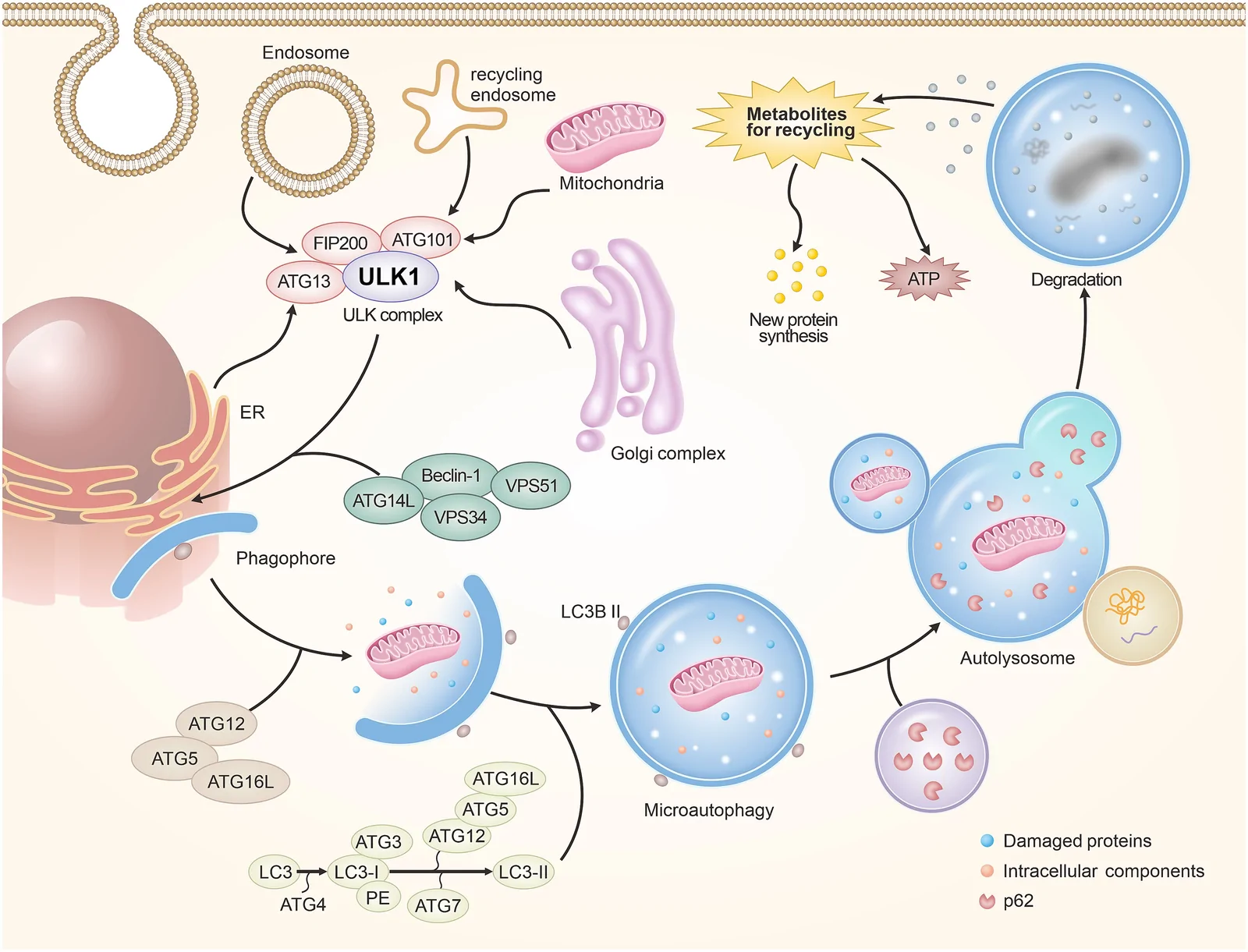
The process of autophagy in mammalian cells requires the coordinated participation of ATG proteins. The ULK1 complex is involved in the induction of phagophore formation, while the Beclin-1-VPS34 complex facilitates nucleation. The ATG12-ATG5-ATG16L complex and LC3-PE mediate the elongation and expansion of the autophagosome. *ATG* autophagy-related, *ULK1* Unc-51-like kinase 1, *FIP200* focal adhesion kinase family-interacting protein of 200 kDa, *VPS* vacuolar protein sorting, *LC3* microtubule-associated protein 1 light chain 3, PEphosphatidylethanolamine, *ATP* adenosine triphosphate

The successful completion of autophagic flux requires precise coordination among multiple autophagy-related (ATG) genes and proteins [27] (Figure 2). The ULK1 complex, consisting of ULK1, ATG13, FIP200, and ATG101, is essential for initiating autophagy; ULK1 itself is a serine/threonine kinase whose deficiency impairs autophagy initiation [28]. Under nutrient-rich conditions, the mammalian target of rapamycin (mTOR) pathway suppresses ULK1 activity, while nutrient deprivation or stress conditions inhibit mTOR and activate AMP-activated protein kinase (AMPK), thus triggering autophagy via ULK1 activation. The activated ULK1 complex phosphorylates Beclin-1, which interacts with ATG14, VPS15, and VPS34 to form the class III phosphatidylinositol 3-kinase (PI3KIII) complex, mediating the nucleation and formation of the phagophore membrane [29].

Following nucleation, two ubiquitin-like conjugation systems facilitate autophagosome expansion: the ATG12–ATG5–ATG16L complex and the LC3 lipidation system [30]. LC3 precursor protein is cleaved by ATG4 to form LC3-I, which is subsequently lipidated to LC3-II through reactions mediated by ATG7 and ATG3. LC3-II is specifically incorporated into autophagosomal membranes and serves as a widely used marker for monitoring autophagy initiation and progression [31, 32]. Meanwhile, the ATG12–ATG5–ATG16L complex enhances the integration of LC3-II into autophagosome membranes, accelerating autophagosome formation.

The autophagy adaptor protein sequestosome 1 (p62/SQSTM1) contains a ubiquitin-binding domain that recognizes ubiquitinated misfolded proteins and damaged organelles. Through its interaction with LC3-II, p62/SQSTM1 selectively delivers substrates into autophagosomes for degradation [33, 34]. Autophagy-related genes play a crucial role in maintaining autophagic flux [35], and genetic defects can lead to autophagy impairment. Studies have shown that in neurons following SCI, impaired autophagic flux results in pathological accumulation of dysfunctional autophagosomes [36, 37]. To better assess autophagic activity, markers such as LC3-II, Beclin-1, and p62/SQSTM1 are commonly used [38]. However, interpretation of these markers requires caution. Increased LC3-II and Beclin-1 levels together with decreased p62/SQSTM1 levels may suggest enhanced autophagic activity, whereas the opposite pattern is more consistent with autophagic flux blockade. Importantly, increased LC3-II alone does not necessarily indicate autophagy activation, because it may reflect either enhanced autophagosome formation or impaired autophagosome clearance due to lysosomal dysfunction. Recent work has further emphasized that restoration of autophagic flux, rather than simple accumulation of LC3-II, is a critical determinant of neuroprotection after SCI. For example, exendin-4 has been reported to improve recovery by reducing lysosomal membrane permeability and restoring lysosomal function and autophagic flux, while more recent studies have linked defective microglial autophagy after SCI to TREM2-associated lysosomal membrane permeabilization [39, 40]. Therefore, autophagic flux should be evaluated by integrating LC3-II and p62/SQSTM1 changes with lysosomal status and, where applicable, flux-monitoring approaches, rather than relying on any single marker alone.

The impact of autophagic flux varies significantly depending on the type and severity of SCI. In models of spinal cord hemisection or complete transection, autophagic flux is notably enhanced, exhibiting robust cellular protective effects and facilitating clearance of damaged cellular components [41, 42]. Conversely, severe spinal cord contusion injuries disrupt autophagic flux, characterized by pathological accumulation of undegraded autophagosomes, exacerbating cellular damage [43, 44]. In spinal cord compression injury models, autophagic flux shows variable responses depending on injury severity. Moderate compression injuries typically enhance autophagic flux, exerting a protective effect. However, severe compression injuries often impede autophagic flux significantly, aggravating injury outcomes [45, 46]. However, in ischemia–reperfusion (I/R) injury models, autophagic flux appears to show temporal dynamics that are not directly correlated with ischemic duration. Studies have consistently reported enhanced autophagic flux during the reperfusion phase, possibly reflecting a compensatory response to oxidative stress and inflammatory damage induced by reperfusion [47–49]. Nevertheless, sustained or excessive autophagic activation may become detrimental, potentially triggering autophagic cell death. Therefore, precise modulation of autophagic flux is crucial.

Effective autophagic flux critically depends on lysosomal integrity and function. Lysosomal membrane permeabilization, lysosomal enzyme dysfunction, or abnormal lysosomal biogenesis can severely disrupt autophagic flux, triggering ER stress and neuronal apoptosis, profoundly impairing tissue repair following SCI [50]. Lysosomal dysfunction-induced autophagic flux disruption and ER stress can trigger motor neuron apoptosis, thereby impairing tissue repair following SCI [13].

In conclusion, autophagic flux plays a multifaceted role in SCI pathology, involving complex regulatory mechanisms operating across multiple levels. Although the precise regulatory pathways of autophagic flux remain incompletely understood, further elucidation of these mechanisms across various injury models will facilitate the development of targeted therapeutic strategies aimed at optimizing autophagy, potentially improving clinical outcomes for SCI patients.

### Autophagy-related signaling pathways in SCI

Autophagy after SCI is regulated by multiple upstream signaling pathways that converge on key functional nodes, including ULK1-dependent initiation, class III PI3K/VPS34-mediated phagophore nucleation, LC3 lipidation, lysosomal biogenesis, and autophagic flux completion. These pathways should not be interpreted simply as linear activators or inhibitors of autophagy, because their biological effects depend on injury stage, injury severity, cellular context, and autophagic flux status. To improve mechanistic clarity, this section is organized into five regulatory modules: mTOR-centered regulation, Transcription factor EB (TFEB)-mediated lysosomal biogenesis, mitogen-activated protein kinase (MAPK)/extracellular signal-regulated kinase 1/2 (ERK1/2) signaling, inflammation-related signaling, and stress-responsive or emerging regulators.

#### mTOR-centered regulation of autophagy initiation and flux: PI3K/Akt/mTOR, AMPK/mTOR, and ULK1

The mTOR is a central regulatory hub involved in autophagy modulation after SCI. mTOR forms two functionally distinct complexes, mTORC1 and mTORC2, among which mTORC1 is most directly involved in autophagy initiation [51]. Under nutrient-rich conditions, mTORC1 phosphorylates ULK1 at Ser757, thereby inhibiting ULK1 complex formation and suppressing autophagy initiation. In contrast, nutrient deprivation, energy stress, or pharmacological mTOR inhibition reduces mTORC1 activity, allowing ULK1 activation and phosphorylation of downstream autophagy-initiation components, including ATG13 and FIP200 [52]. Thus, the mTORC1–ULK1 axis functions as a key molecular switch linking stress signals to autophagy initiation [52, 53].

The PI3K/Akt/mTOR pathway is a major upstream regulator of mTORC1 and controls cell growth, survival, apoptosis, and autophagy [54, 55]. In SCI, inhibition of this pathway can promote autophagic activity and neuroprotection. For example, melatonin inhibits PI3K/Akt/mTOR signaling, enhances neuronal autophagy, suppresses apoptosis, and improves motor function after SCI [56]. Similarly, PI3K inhibition decreases Akt and mTOR phosphorylation while increasing LC3-II expression in injured spinal cord neurons, suggesting enhanced autophagy initiation or autophagic activity [57]. However, the effect of this pathway is context-dependent. Basic fibroblast growth factor activates PI3K/Akt/mTOR signaling, suppresses excessive autophagy, and protects motor neurons [58]. These findings suggest that PI3K/Akt/mTOR signaling may be protective either through autophagy induction or suppression, depending on whether autophagy is insufficient, excessive, or functionally impaired.

AMPK is another major regulator of the mTOR-centered autophagy network. AMPK is activated by an increased AMP/ATP ratio during energy stress [59]. After SCI, mitochondrial dysfunction and metabolic disturbance may activate AMPK, which can promote autophagy by inhibiting mTORC1 through TSC2 or Raptor and by phosphorylating ULK1 at activation sites such as Ser317, Ser467, and Ser777 [60]. Recent evidence also suggests that AMPK–ULK1 regulation may be more context-dependent than previously appreciated under different forms of energy stress [61, 62]. In SCI models, metformin improves motor function, reduces p62/SQSTM1 and ubiquitinated protein accumulation, activates AMPK, and inhibits mTOR signaling, suggesting restoration of autophagic flux [45]. Similar protective effects have been reported for zinc [63] and resveratrol [64]. Collectively, mTOR-centered signaling integrates growth factor, nutrient, and energy-stress cues to regulate autophagy after SCI.

#### TFEB-mediated lysosomal biogenesis and autophagic flux completion

Autophagy initiation alone is not sufficient for effective cytoprotection; completion of autophagic flux also requires autophagosome–lysosome fusion and lysosomal degradation. TFEB is a master regulator of lysosomal biogenesis and autophagy-related gene expression [65]. Under basal conditions, mTORC1 phosphorylates TFEB and restricts its nuclear translocation [66]. When mTORC1 activity is suppressed, TFEB translocates into the nucleus and activates transcriptional programs involved in lysosomal biogenesis, autophagosome maturation, and substrate degradation [67, 68].

This mechanism is particularly relevant to SCI because impaired lysosomal function can cause incomplete autophagic flux and pathological accumulation of undegraded autophagosomes. Therefore, enhancing lysosomal capacity may be more beneficial than merely increasing autophagosome formation. Netrin-1 [69] and betulinic acid [70] have been reported to upregulate autophagy, reduce neuronal apoptosis, and promote functional recovery after SCI, partly through AMPK/mTOR/TFEB-mediated lysosomal biogenesis and autophagic flux restoration. Thus, TFEB-centered regulation highlights the need to evaluate autophagy as a dynamic process that includes both initiation and completion.

#### MAPK/ERK1/2 signaling and context-dependent autophagy regulation

MAPK signaling, particularly the ERK1/2 pathway, is closely associated with autophagy regulation after SCI. MAPK/ERK1/2 signaling can interact with mTOR-related pathways and influence neuronal survival, apoptosis, inflammation, and autophagy. However, its relationship with autophagy remains context-dependent. Bisperoxovanadium activates ERK1/2 signaling, promotes autophagy, inhibits apoptosis, and reduces neuronal loss [71]. In contrast, *Tripterygium wilfordii* treatment decreases ERK1/2 phosphorylation while enhancing autophagy and reducing neuronal cell death after SCI [72]. Furthermore, miR-103 overexpression reduces p-ERK and p-MAPK levels, suppresses autophagy, and exerts protective effects in SCI rats [73].

These divergent findings suggest that MAPK/ERK1/2 signaling should not be classified as purely autophagy-promoting or autophagy-inhibiting. Differences in injury model, treatment timing, intervention type, cell type, and baseline autophagic flux may explain why apparently opposite pathway changes can still produce neuroprotective outcomes.

#### Inflammation-related autophagy regulation: NF-κB, NLRP3 inflammasome, and JAK/STAT

Autophagy is closely connected with inflammatory signaling after SCI. Nuclear factor-κB (NF-κB) is a central transcriptional regulator of inflammatory responses and contributes to neuroinflammation, glial activation, and secondary tissue damage [74]. Autophagy may interact with NF-κB signaling bidirectionally: inflammatory stimulation can alter autophagic activity, whereas autophagy can suppress excessive inflammation by promoting the clearance of damaged mitochondria and limiting inflammasome-related inflammatory cascades [75, 76].

The NLRP3 inflammasome is another important inflammatory node regulated by autophagy. NLRP3 activation promotes caspase-1 activation and IL-1β maturation, thereby amplifying inflammation-driven secondary injury. Autophagy may inhibit NLRP3 activation by preserving mitochondrial integrity, reducing mitochondrial ROS production, limiting mitochondrial DNA release, and facilitating degradation of inflammasome-related components. In SCI, Pien–Tze–Huang promotes autophagy via the AMPK/mTOR/ULK1 pathway and suppresses NLRP3 inflammasome-mediated neuroinflammation, whereas salidroside regulates microglial autophagic flux and polarization through AMPK/mTOR signaling [77, 78]. Zinc also regulates the NLRP3 inflammasome in SCI-induced microglia through autophagy- and ubiquitination-related mechanisms [79]. In addition, JAK/STAT signaling may participate in cytokine-mediated regulation of autophagy and apoptosis after SCI [80]. These findings suggest that autophagy modulation may protect the injured spinal cord not only through neuronal quality control, but also through reshaping the inflammatory microenvironment.

#### Stress-responsive and emerging regulators: Keap1/Nrf2/ARE, HIF-1, and Notch

SCI is characterized by oxidative stress, hypoxia, mitochondrial dysfunction, and extracellular matrix remodeling, all of which can influence autophagy. The Keap1/Nrf2/ARE pathway links oxidative stress to autophagy regulation [81]. Under oxidative stress, Nrf2 dissociates from Keap1 and translocates into the nucleus, where it activates antioxidant response element (ARE)-driven transcription of cytoprotective genes. Autophagy intersects with this pathway through p62/SQSTM1. When autophagic flux is blocked, p62/SQSTM1 may accumulate and interact with Keap1, thereby facilitating Nrf2 activation [82, 83]. However, p62/SQSTM1 accumulation may also indicate impaired autophagic degradation; therefore, Nrf2 activation should be interpreted together with autophagic flux status.

Hypoxia-inducible factor-1 (HIF-1) signaling may regulate autophagy after SCI, particularly under ischemic or hypoxic conditions [47]. HIF-1-related mechanisms can influence mitochondrial metabolism, mitophagy, and adaptive responses to oxygen deprivation. Notch signaling has also been implicated in autophagy regulation during SCI [84]. Experimental evidence suggests that autophagy may promote axonal regeneration by limiting Notch signaling in an age-dependent manner, indicating a possible connection between autophagy, developmental signaling, and repair capacity after injury. These emerging pathways indicate that autophagy after SCI is not controlled solely by classical mTOR- or AMPK-dependent mechanisms, but also by broader stress-responsive and developmental signaling networks.

Overall, the therapeutic significance of these signaling pathways depends less on whether they simply activate or inhibit autophagy, and more on whether they restore functional autophagic flux and cellular homeostasis. Inhibition of PI3K/Akt/mTOR signaling or activation of AMPK/mTOR signaling may be beneficial when autophagic activity is insufficient or flux is impaired, whereas suppression of excessive autophagy may be protective when sustained autophagic activation contributes to neuronal loss or autophagic cell death. Therefore, pathway-targeted interventions should be interpreted according to injury stage, severity, cell type, and flux status. From a translational perspective, strategies aimed at normalizing autophagic flux and lysosomal function may have greater value than those that merely increase or decrease autophagy markers [15, 45, 58, 70]. The major signaling pathways and regulatory nodes involved in autophagy after SCI are summarized in Table 1.

**Table 1:** Major signaling pathways and regulatory nodes involved in autophagy after SCI

Regulatory pathway or node	Key components	Autophagy-related mechanism and implication in SCI	References
PI3K/Akt/mTOR	PI3K, PIP3, PDK1, Akt, mTORC1	Inhibition of PI3K/Akt/mTOR signaling may promote autophagy initiation, whereas pathway activation may suppress excessive autophagy. Its therapeutic effect is context-dependent and depends on baseline flux status.	[56–58]
AMPK/mTOR/ULK1	AMPK, TSC2, Raptor, mTORC1, ULK1	AMPK activation inhibits mTORC1 and promotes ULK1-dependent autophagy initiation, linking metabolic stress to autophagic flux regulation and damaged-organelle clearance.	[60, 63, 64]
AMPK/mTOR/TFEB	AMPK, mTORC1, TFEB, lysosomal genes	mTORC1 suppression promotes TFEB nuclear translocation, enhancing lysosomal biogenesis and autophagic flux completion rather than merely increasing autophagosome formation.	[66–68, 70]
MAPK/ERK1/2/mTOR	Ras, Raf, MEK1/2, ERK1/2, mTORC1	ERK1/2-related signaling may either enhance or suppress autophagy depending on injury model, intervention timing, cell type, and baseline flux status.	[71–73]
NF-κB/NLRP3 inflammasome	NF-κB, IKKβ, IκB, NLRP3, caspase-1, IL-1β	Autophagy may limit neuroinflammation by clearing damaged mitochondria and suppressing NF-κB- or NLRP3-related inflammatory cascades in microglia/macrophages.	[74–79]
Keap1/Nrf2/ARE	p62/SQSTM1, Keap1, Nrf2, ARE	p62/SQSTM1 accumulation may disrupt Keap1-mediated Nrf2 inhibition and activate antioxidant responses, but should be interpreted together with autophagic flux status.	[81–83]
JAK/STAT	JAK2, STAT3	JAK/STAT signaling may connect cytokine-mediated inflammation, apoptosis, and autophagy regulation during secondary injury after SCI.	[80]
HIF-1	HIF-1α, hypoxia-responsive genes	HIF-1-related signaling may regulate autophagy and mitophagy under ischemic or hypoxic conditions, linking oxygen deprivation to mitochondrial adaptation.	[47]
Notch	Notch receptors, downstream transcriptional targets	Autophagy may interact with Notch signaling and support axonal regeneration by limiting Notch activity in specific repair contexts.	[84]

*SCI* spinal cord injury, *ERK* extracellular signal-regulated kinase, *IL* interleukin, *HIF-1* hypoxia-inducible factor 1

### Phase-dependent roles of autophagy in SCI

Autophagy after SCI is highly dynamic and varies across different stages of injury. Therefore, in addition to its involvement in specific pathological processes, autophagy should also be interpreted from a phase-dependent perspective. In the acute phase, autophagy is closely associated with oxidative stress, mitochondrial damage, and lysosomal dysfunction. In the subacute and intermediate phases, its role becomes increasingly linked to neuroinflammation, glial activation, and tissue remodeling. In the chronic phase, autophagy may further influence axonal remodeling and repair-related processes.

#### Acute phase: oxidative stress and autophagic dysfunction

Oxidative stress refers to an imbalance between ROS and endogenous antioxidant defenses. ROS, a group of highly reactive oxygen-containing species, including superoxide and hydrogen peroxide, are generated primarily through mitochondrial oxidative metabolism [85]. ROS levels can substantially affect cellular functions. At low levels, ROS participate in regulating various cellular signaling pathways and physiological functions. However, excessive ROS production leads to lipid peroxidation and the oxidation of proteins and DNA, triggering apoptosis, disrupting cell membranes, and exacerbating the progression of neurological diseases [86]. Extensive research has shown that ROS and the oxidative stress they induce play crucial roles in secondary injury following SCI [87]. Pathological changes such as edema, hemorrhage, and hypoxia lead to mitochondrial dysfunction and activate ROS-producing enzymes such as xanthine oxidase, causing excessive ROS production [88]. As endogenous antioxidant capacity is exceeded, ROS accumulate at the injury site, potentially leading to lysosomal dysfunction and the inhibition of autophagy. Autophagy, an essential cellular self-cleaning mechanism, should help eliminate damaged cellular components, but it is suppressed by oxidative stress following SCI, thereby worsening the injury.

Transcription factor E3 (TFE3), a member of the MiT/TFE family, plays an essential role in regulating lysosomal function and oxidative metabolism. Research indicates that TFE3 levels are negatively correlated with ROS levels, and increasing TFE3 expression can reduce ROS accumulation and improve autophagic flux. This suggests that modulating TFE3 could help alleviate ROS-induced damage and restore autophagic function. However, ROS can have dual effects on autophagic flux. First, ROS can directly influence autophagy through redox-sensitive mediators like ATG4; second, ROS can indirectly promote autophagy through upstream pathways such as AMPK [89]. These findings highlight the complex and context-dependent regulation of autophagy by ROS, suggesting that ROS can either activate or inhibit autophagic flux depending on their levels and the cellular environment.

**Figure 3:**
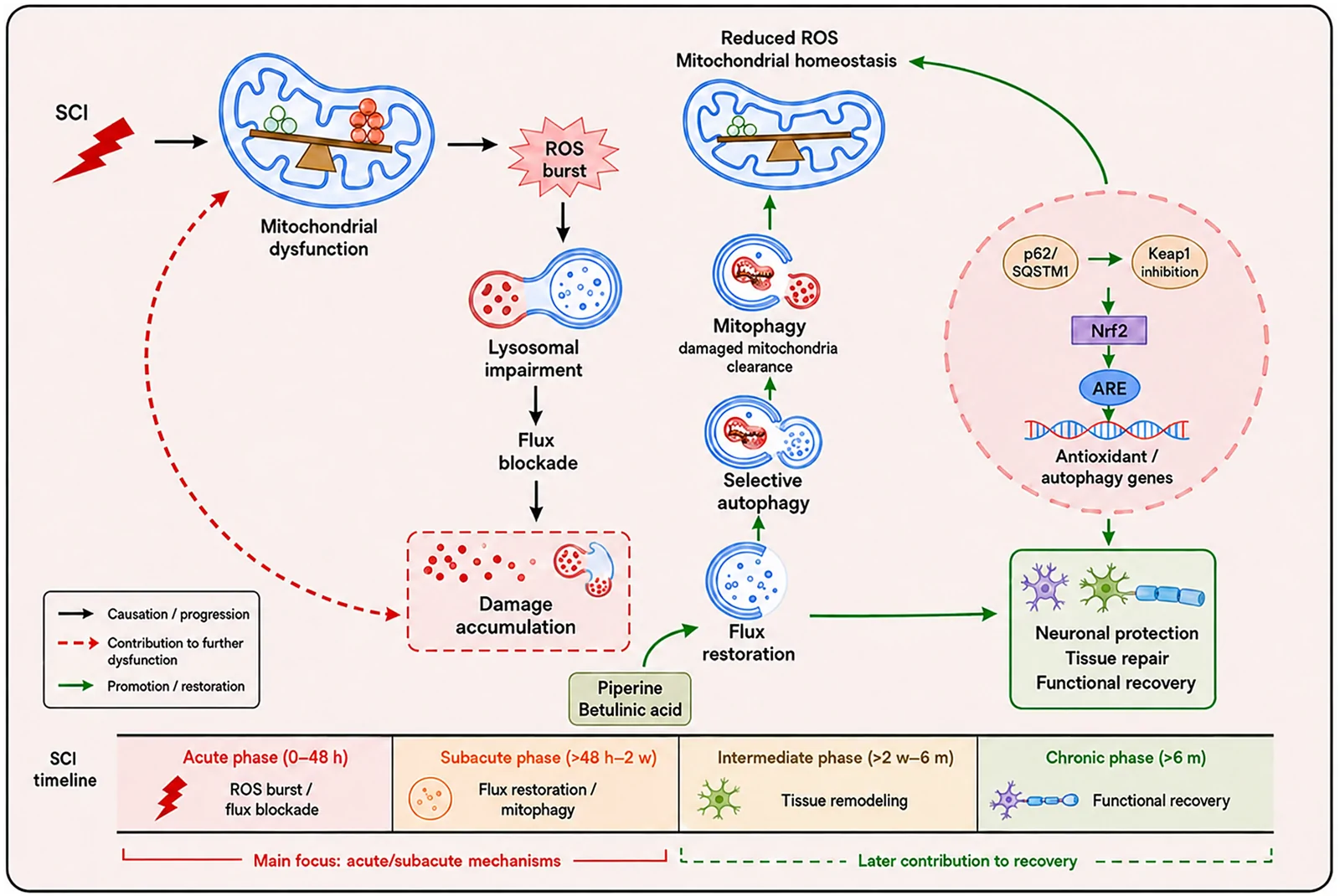
Phase-related regulation of ROS homeostasis by autophagic flux and mitophagy after SCI. During the acute phase (0–48 h), SCI-induced mitochondrial dysfunction promotes ROS burst, lysosomal impairment, autophagic flux blockade, and damage accumulation. During the subacute phase (>48 h–2 w), restoration of autophagic flux promotes selective autophagy, particularly mitophagy, thereby clearing damaged mitochondria, reducing mitochondrial ROS, and restoring mitochondrial homeostasis. The p62/SQSTM1–Keap1–Nrf2/ARE axis further supports antioxidant and autophagy-related gene expression. These early mechanisms may contribute to tissue remodeling in the intermediate phase and functional recovery in the chronic phase. Black arrows indicate pathological progression, red dashed arrows indicate contribution to further dysfunction, and green arrows indicate protective or restorative processes

Activating autophagy has been shown to reduce oxidative stress and decrease ROS production in spinal cord neurons following injury [90] (Figure 3). Piperine, a natural active alkaloid extracted from black pepper, has been found to reduce ROS accumulation by enhancing autophagy, thereby alleviating SCI-induced neurodegeneration [91]. This mechanism emphasizes the importance of autophagy enhancement to reduce ROS levels and mitigate oxidative stress-induced cellular damage. Mitophagy, a specialized form of selective autophagy, removes damaged mitochondria and thereby supports cellular homeostasis. Restoration of autophagic flux after SCI may promote mitophagy, reduce ROS accumulation, and suppress necroptosis [70]. This research underscores the importance of mitophagy in SCI recovery, as it helps eliminate damaged mitochondria and reduces oxidative stress and cell death triggered by defective mitochondria.

Nrf2 is a key regulator of the cellular antioxidant response to oxidative stress. It is negatively regulated by Keap1, and upon oxidative stress, Keap1 is degraded, allowing Nrf2 to translocate to the nucleus, where it binds to ARE and activates the transcription of various antioxidant genes. The activation of the Nrf2 pathway has been shown to promote mitophagy, suppress ROS-induced apoptosis, and regulate mitochondrial function [92, 93]. After severe SCI, the blockade of autophagic flux leads to the accumulation of p62/SQSTM1, which binds with Keap1, displacing Nrf2 [81]. The displaced Nrf2 enters the nucleus, where it activates the transcription of antioxidant genes, suppressing the expression of oxidative stress-related proteins and improving the recovery of motor neurons after SCI [94]. These findings indicate that the Keap1/Nrf2/ARE signaling pathway plays a pivotal role in autophagy regulation following SCI and represents a potential therapeutic target in SCI.

In conclusion, restoration of autophagic flux and activation of mitophagy may reduce ROS accumulation, whereas flux blockade may trigger compensatory p62/SQSTM1-Nrf2-related responses but also reflects impaired autophagic degradation. Modulating oxidative stress through autophagy-dependent regulation of the Keap1/Nrf2/ARE pathway may offer a potential therapeutic approach for SCI. Further research into the interactions between autophagy and oxidative stress, as well as the regulation of these signaling pathways, will be essential for developing targeted treatments for SCI. Understanding how different levels of ROS affect autophagy regulation will also provide deeper insights into potential therapies, making it a promising area for future exploration in SCI treatment.

#### Subacute and intermediate phases: neuroinflammation and tissue remodeling

Neuroinflammation is a major component of secondary SCI and becomes particularly prominent during the subacute and intermediate phases, when activated resident glial cells and infiltrating immune cells collectively shape tissue remodeling and injury progression. Hours after the primary injury, resident cells (including microglia and astrocytes) and infiltrating macrophages, neutrophils, and lymphocytes are activated and recruited to the injury site [95]. These cells release inflammatory factors such as TNF-α, IL-1α, IL-1β, and IL-6, which amplify the neuroinflammatory response and exacerbate SCI progression [96]. During these stages, autophagy becomes increasingly important not only as an intracellular degradative process, but also as a regulator of the inflammatory microenvironment and tissue remodeling. Thus, the subacute and intermediate phases represent a critical window in which autophagy may influence both injury propagation and subsequent repair. The cell-type-specific role of autophagy in microglia/macrophages is discussed in detail below.

#### Chronic phase: axonal remodeling and repair-related processes

In the chronic phase, SCI pathology is increasingly characterized by persistent glial responses, incomplete circuit reconstruction, and limited structural repair. At this stage, the role of autophagy appears to be more closely linked to long-term maintenance of neuronal integrity, axonal remodeling, and repair-related processes. Although the detailed mechanisms are discussed below from a cell-type-specific perspective, current evidence suggests that autophagy may contribute to chronic-stage recovery by supporting cytoskeletal stability, cellular homeostasis, and tissue remodeling.

Overall, the interplay between autophagy and microtubule dynamics in SCI recovery is an area of growing interest. The stabilization of microtubules through acetylation and autophagy activation provides a promising therapeutic strategy to promote axonal regeneration and functional recovery following SCI. Future studies focused on understanding the molecular mechanisms of autophagy regulation and microtubule dynamics, along with the development of targeted therapies, are essential to improving outcomes in SCI treatment [97, 98].

### Cell-type-specific roles of autophagy in SCI

In addition to its phase-dependent roles, autophagy also exhibits marked cellular heterogeneity after SCI. Its effects differ substantially among neurons, microglia/macrophages, astrocytes, and oligodendrocytes, which may partly explain the divergent outcomes reported in different studies. Therefore, a cell-type-specific framework is necessary to better understand the context-dependent role of autophagy in SCI.

#### Neurons

In neurons, autophagy is closely linked to intracellular quality control, cytoskeletal stability, and axonal repair after SCI. Because mature central nervous system (CNS) neurons have limited regenerative capacity, restoration of axonal growth depends heavily on microtubule remodeling and the maintenance of structural integrity. Microtubules are polymers composed primarily of α- and β-tubulin dimers and interact with microtubule-associated proteins. These microtubules play an essential role in determining the position and function of cellular structures and organelles [99, 100]. During axon formation, microtubule dynamics and post-translational modifications, are crucial for axon development and the polarization of neurons [101, 102].

Tubulin acetylation, a post-translational modification, serves as an important marker for microtubule stability. Previous studies have shown that acetylation of microtubules stabilizes the microtubule system of neuronal axons, reduces the scar area following SCI, and promotes axonal regeneration [103, 104]. Autophagy-mediated axonal regeneration after SCI is closely linked to the microtubule network. Retrograde transport of autophagosomes and their fusion with lysosomes are dependent on the stability of the microtubule system [105]. Research has demonstrated that Schwann cell-derived exosomes can upregulate autophagy by inhibiting the Akt/mTOR signaling pathway, which further enhances tubulin acetylation and microtubule polymerization, thus promoting axonal growth and repair [106]. In addition to these findings, studies have also highlighted the role of liraglutide in SCI recovery. Liraglutide can stimulate autophagy, reducing apoptosis and improving functional recovery while simultaneously increasing tubulin acetylation after SCI [107]. This suggests that autophagy not only serves as a neuroprotective mechanism but also directly contributes to the stabilization of microtubules, which is crucial for the restoration of axonal integrity.

A critical protein in the regulation of microtubule stability and dynamics is SCG10, a microtubule-destabilizing protein. Autophagy contributes to microtubule stabilization and axonal regeneration after SCI by promoting SCG10 degradation [108]. However, some studies have also suggested that SCG10 overexpression may facilitate the extension and elongation of cellular protrusions by increasing microtubule dynamics [109]. These apparently conflicting findings suggest that the role of SCG10 is highly context-dependent rather than simply beneficial or detrimental. A plausible explanation is that transient microtubule destabilization may support early growth cone remodeling and axonal sprouting, whereas subsequent microtubule stabilization may be required for sustained axonal elongation and structural maintenance. To resolve this controversy, future studies should analyze SCG10-autophagy interactions in a stage-specific and cell-specific manner, distinguish autophagy induction from impaired autophagic flux, and evaluate both structural endpoints (such as sprouting and axonal extension) and functional recovery. Such a framework may help clarify whether SCG10 exerts distinct effects at different phases of SCI and may identify a more precise therapeutic window for targeting the SCG10-autophagy axis.

Histone deacetylases (HDACs), a group of enzymes involved in the regulation of chromatin structure and gene expression, also play a pivotal role in microtubule dynamics. HDACs can specifically catalyze the deacetylation of non-histone substrates, including tubulin proteins, thereby influencing a variety of physiological and pathological processes. Inhibition of HDAC6, one of the key deacetylases involved in microtubule regulation, has been shown to increase the acetylation of tubulin, restore autophagic flux, and promote functional recovery following SCI [110]. This highlights the potential therapeutic value of regulating autophagy and microtubule dynamics in promoting axonal regeneration after SCI.

The intricate relationship between autophagy and microtubule dynamics suggests that autophagy does not only play a role in cellular housekeeping and damage repair but also modulates the cytoskeletal network, which is essential for axonal growth. Given that microtubule stability is critical for the formation of growth cones and axonal outgrowth, it is likely that modulating autophagy to either enhance or stabilize microtubules could have significant therapeutic benefits in SCI repair [111–113].

Moreover, autophagy has been implicated in the clearance of damaged or dysfunctional cellular components, including the regulation of mitochondrial function. Mitophagy, the selective autophagic degradation of damaged mitochondria, is essential for maintaining neuronal health after SCI [50, 114]. Mitochondrial dysfunction is one of the major contributors to oxidative stress and cell death in the injured spinal cord, and promoting mitophagy could reduce mitochondrial damage and enhance cellular recovery [115, 116]. This underscores the broader role of autophagy in protecting neuronal cells during SCI recovery, beyond simply its effects on microtubule dynamics.

Several signaling pathways are involved in regulating the autophagic process during SCI recovery, particularly the PI3K/Akt/mTOR and AMPK pathways. These pathways control autophagy in response to various cellular stresses and energy demands [55]. As mentioned earlier, the Akt/mTOR pathway can inhibit autophagy, whereas autophagy activation through AMPK has been associated with improved axonal regeneration and functional recovery [43, 117]. Thus, targeting these pathways for modulating autophagy could be a viable therapeutic strategy for enhancing microtubule stability and supporting axonal regrowth in SCI. Consistent with this view, a recent study showed that inhibition of CSPG–PTPσ can activate autophagy flux and lysosome fusion, thereby facilitating axonal and synaptic reorganization and improving functional recovery after SCI.

In addition, the role of Schwann cells and their secreted factors, including exosomes, in modulating both autophagy and axonal regeneration is an exciting area of investigation. Schwann cells are known to support axonal growth and repair in peripheral nerve injury, and recent research suggests that Schwann cell-derived exosomes may facilitate this process by regulating autophagy and promoting microtubule polymerization [106, 118, 119]. This has important implications for enhancing spinal cord repair, as Schwann cell-based therapies may also be applicable to CNS injuries.

#### Microglia/macrophages

Growing evidence links autophagy to microglial inflammation. The activation or inhibition of autophagy is associated with either anti-inflammatory or pro-inflammatory effects. Activation of autophagy can reduce the number of activated microglia and inhibit the secretion of pro-inflammatory cytokines such as TNF-α, IL-1β, and IL-6, thereby alleviating neuroinflammation [120]. More recent studies further support the idea that autophagy modulation can reshape microglial inflammatory programs after SCI. Combined human adipose-derived stem cell (hADSC) transplantation and green light therapy improved recovery in part through restored microglial autophagy, and zinc ion treatment was reported to facilitate post-SCI metabolic recovery by activating microglial mitophagy. Research by Goldshmit *et al.* also confirmed that after the injection of the autophagy inducer rapamycin, activation of M1 microglia at the injury site was reduced, inflammation decreased, and neuronal survival was promoted [121]. Autophagy activation can also regulate the polarization of microglia from M1 to M2, thereby protecting neurons from apoptosis [78]. Progranulin enhances microglial autophagy by protecting lysosomal function, promotes M2 polarization of microglia, exerts anti-inflammatory effects, and ultimately improves neurological function after acute SCI [122]. Conversely, inhibition of autophagy may lead to heightened microglial activation. Li *et al.* further confirmed through transcriptomic, cellular, and molecular studies that autophagy deficiency exacerbates neuroinflammation, resulting in poorer functional recovery and increased tissue damage [14]. Regulation of autophagy can affect microglial inflammatory responses, and conversely, a pro-inflammatory environment may also regulate autophagic activity, as shown by the increased expression of LC3 and p62/SQSTM1 in lipopolysaccharide (LPS)-induced microglial inflammation models [78]. In conclusion, inhibiting the inflammatory response through the regulation of autophagy may provide a therapeutic approach for treating SCI.

Autophagy may alleviate neuroinflammation by mediating the degradation of the NLRP3 inflammasome. The NLRP3 inflammasome is a multiprotein complex found in microglia within the CNS, and it is notably expressed and activated after SCI [123, 124]. NLRP3 responds to trauma by regulating caspase-1 activation and the maturation of IL-1β, thereby promoting inflammation-driven neuropathology following SCI [125, 126]. Activation of cannabinoid receptor-2 increases the ubiquitination of the NLRP3 inflammasome, a mechanism related to the promotion of autophagy [127]. Zinc regulates the NLRP3 inflammasome in SCI-induced microglia through both autophagy and ubiquitination, thus providing neuroprotective effects [79]. Although there is a link between autophagy and the NLRP3 inflammasome, the regulatory mechanisms involved in SCI remain incompletely understood. Therefore, further investigation into these mechanisms could contribute to the discovery of new therapeutic approaches for SCI.

Autophagy can also regulate the inflammatory response in SCI through the NF-κB pathway. The NF-κB signaling pathway plays a crucial role in the specific regulation of the inflammatory cascade and the precise control of the inflammatory response. When stimuli activate NF-κB, the activated NF-κB translocates into the nucleus, where it regulates the transcription of genes involved in inflammation, such as inflammatory cytokines. Inhibiting the NF-κB pathway can reduce the SCI-induced inflammatory response and improve functional recovery [128]. There seems to be a broad and complex regulatory relationship between the NF-κB signaling pathway and autophagy [129]. NF-κB can promote autophagy by inducing the expression of autophagy-related proteins. In turn, autophagy can suppress the NF-κB pathway by downregulating the IKK complex [130]. Guo *et al.* found that after treating SCI mice with granulocyte colony-stimulating factor (G-CSF), autophagy activation was accelerated, and the NF-κB signaling pathway was inhibited. They concluded that timely use of G-CSF after SCI could promote autophagy and protect neuronal structures by inhibiting the NF-κB pathway [131]. Omega-3 free fatty acids enhance autophagy by degrading inflammatory complexes, thereby reducing inflammation. They also inhibit NF-κB activation, preventing the initiation of inflammation [132]. The NF-κB signaling pathway may be a key component in the SCI–autophagy–inflammation axis, making it a promising target for further research.

The PI3K/AKT/mTOR signaling pathway is a major regulator of autophagy and is also involved in the regulation of autophagy-related inflammatory pathways. Based on a series of *in vitro* and *in vivo* studies, Zhang *et al.* revealed that inhibition of the PI3K/AKT/mTOR pathway enhances microglial autophagy, thereby promoting the polarization of microglia toward the M2 phenotype [133]. Peripheral macrophage-derived exosomes promote autophagy by inhibiting AKT and mTOR expression, thereby enhancing the anti-inflammatory activity of local microglia. This pathway is an important mechanism for neuronal protection [133]. Moreover, the AMPK/mTOR signaling pathway-mediated autophagy also appears to be involved in the neuroinflammation associated with SCI. Pien–Tze–Huang (PTH), a traditional Chinese medicine, promotes autophagy via the AMPK/mTOR/ULK1 pathway and inhibits microglial activation, thereby suppressing NLRP3 inflammasome-mediated neuroinflammation [77]. Salidroside, a plant extract, regulates microglial autophagic flux and polarization via the AMPK/mTOR pathway [78]. Therefore, agents such as PTH, salidroside, and resveratrol [64], may exert neuroprotective effects through the autophagy-inflammation pathway, suggesting that targeting the autophagy-inflammation axis could be a promising new direction for SCI treatment.

#### Astrocytes

Compared with neurons and microglia/macrophages, the role of autophagy in astrocytes after SCI remains less extensively characterized, but available evidence suggests that it is closely related to reactive astrogliosis, glial scar formation, and regulation of the local reparative microenvironment. After SCI, astrocytes rapidly accumulate around the lesion border, where they can both restrict the spread of inflammatory damage and contribute to a regeneration-inhibitory extracellular environment. In this context, astrocytic autophagy appears to influence not only intracellular homeostasis, but also the balance between protective scar containment and maladaptive scar remodeling. In particular, recent studies have shown that TGF-β-driven astrocyte activation can suppress autophagic flux while promoting secretion of chondroitin sulfate proteoglycans such as neurocan and brevican, suggesting that impaired astrocytic autophagy may contribute to glial scar-associated inhibition of axonal regeneration [15, 95, 96].

Recent evidence further indicates that astrocytic autophagy is associated with phenotypic transformation rather than merely with nonspecific astrocyte activation. A 2023 study linked autophagy-related pathways to A1 astrocyte activation after SCI through the circHIPK2/miR-124-3p/Smad2 axis, while a 2024 study showed that repetitive transcranial magnetic stimulation improved recovery through regulation of a Cx43-autophagy loop. Together, these findings suggest that astrocytic autophagy may influence SCI pathology through at least three interconnected processes: regulation of reactive astrocyte phenotype, control of CSPG-rich scar formation, and modulation of astrocyte-associated intercellular signaling. Future studies should further clarify how astrocytic autophagic flux varies across injury stages and reactive states, and whether targeting astrocyte-specific autophagy can improve the permissiveness of the post-injury microenvironment for neural repair [16, 134].

#### Oligodendrocytes

Autophagy in oligodendrocytes has received less attention than neuronal or microglial autophagy in SCI, but available evidence suggests that it may be critically important for white matter preservation, myelin maintenance, and long-term functional recovery. Oligodendrocytes are highly vulnerable to oxidative stress, excitotoxicity, inflammatory mediators, and trophic deprivation after SCI, and their loss contributes directly to demyelination and chronic axonal dysfunction. The strongest direct evidence comes from a 2018 study showing that conditional deletion of Atg5 in oligodendrocytes after contusive SCI worsened white matter loss and significantly limited locomotor recovery, demonstrating that oligodendroglial autophagy is a necessary cytoprotective mechanism rather than a purely epiphenomenal stress response. This study also suggested that nonspecific manipulation of autophagy at the whole-tissue level may fail to capture the benefit of oligodendrocyte-specific autophagy, highlighting the importance of cell-type-specific interpretation [17].

**Figure 4:**
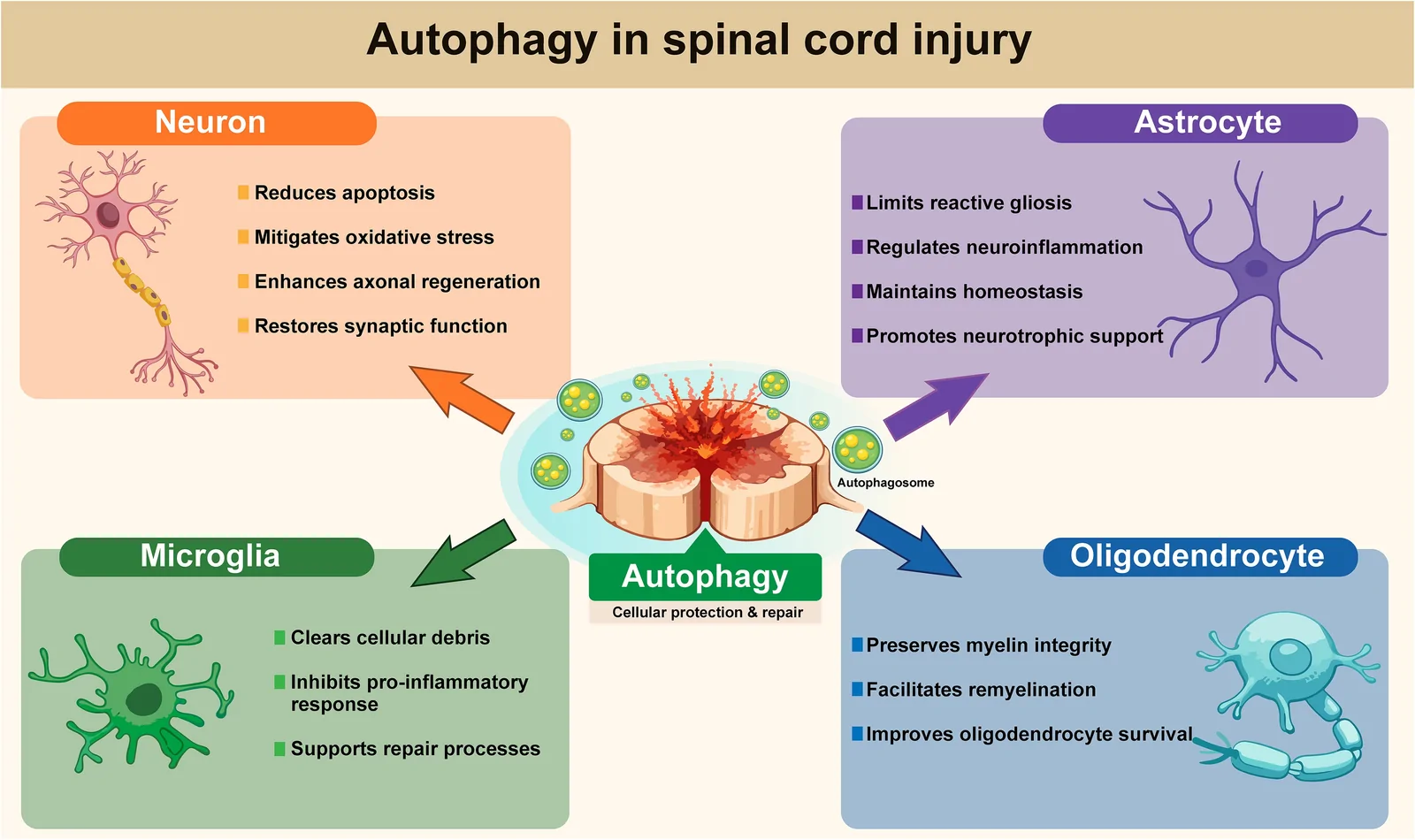
Cell-type-specific heterogeneity of autophagy in spinal cord injury. Autophagy exerts distinct roles in different cell types after spinal cord injury. In neurons, it supports intracellular homeostasis, resistance to oxidative stress and apoptosis, and axonal/synaptic repair. In microglia, it promotes debris clearance and modulates inflammatory responses. In astrocytes, it is involved in reactive gliosis, neuroinflammation, and maintenance of the local reparative microenvironment. In oligodendrocytes, it contributes to myelin integrity, remyelination, and cell survival. Overall, these cell-type-specific effects converge on cellular protection and tissue repair

The relevance of oligodendroglial autophagy is further supported by broader studies of myelinating glia, which indicate that autophagy contributes to oligodendrocyte precursor survival, maturation, myelin homeostasis, and long-term turnover of myelin membranes. These observations are highly relevant to SCI, because chronic disability after injury is strongly influenced by white matter integrity, spared myelin, and the capacity for remyelination. Taken together, current evidence suggests that oligodendrocyte autophagy may affect SCI outcomes by promoting oligodendrocyte survival, preserving white matter integrity, and supporting long-term myelin maintenance and remyelination. Future studies should further determine whether the therapeutic value of oligodendroglial autophagy lies primarily in protecting mature oligodendrocytes, promoting oligodendrocyte precursor cell differentiation, or facilitating myelin turnover during chronic-stage repair [135, 136]. The cell-type-specific heterogeneity of autophagy in SCI, including its distinct roles in neurons, microglia/macrophages, astrocytes, and oligodendrocytes, is summarized in Figure 4.

### Autophagy activators and inhibitors

No curative treatment for SCI is currently available. Currently, surgery and pharmacological interventions are the primary treatment options in clinical practice. Numerous studies have shown that certain drugs can modulate autophagy and exert neuroprotective effects in SCI. We summarize the pharmacological agents reported over the past five years to exert neuroprotective effects through autophagy modulation. Overall, the comparative value of these agents depends less on whether they act as autophagy inducers or inhibitors, and more on whether they restore a beneficial autophagic state in a context-dependent manner. This may explain why agents with apparently opposite effects on autophagy can both show neuroprotective outcomes in SCI models.

#### Drugs that induce or promote autophagy in SCI

Rapamycin is an antifungal drug that inhibits the mTOR pathway and increases autophagosome formation, making it a commonly used autophagy activator [137]. In SCI, rapamycin-activated autophagy exerts protective effects through anti-inflammatory and anti-apoptotic actions [121], inhibition of astrocyte proliferation, and promotion of axonal regeneration. However, rapamycin-mediated autophagy has also been found to inhibit the clearance of ubiquitinated proteins and impair functional recovery [58]. Vascular endothelial growth factor (VEGF) is a secreted mitogen that promotes angiogenesis and revascularization following SCI. VEGF is also considered an effective neurotrophic factor for spinal cord neuron survival. Studies have shown that the application of VEGF165 in young male Wistar rats with SCI can improve the rats’ Basso, Beattie, and Bresnahan (BBB) scores, reduce motor neuron loss, lower the expression of inflammatory factors such as IL-1β, TNF-α, and IL-10, and upregulate the expression of Beclin-1 and LC3B [138]. VEGF165 alleviates SCI by inhibiting inflammation and enhancing autophagic function. Ezetimibe is an antihyperlipidemic drug that lowers cholesterol levels. Ezetimibe can activate autophagy and inhibit apoptosis by modulating the PI3K/AKT/mTOR signaling pathway, thus protecting against SCI [139]. Melatonin, a hormone mainly secreted by the pineal gland, has neuroprotective properties. Melatonin can act on both the PI3K/AKT/mTOR and SIRT1/AMPK pathways to enhance autophagy and reduce apoptosis [56, 140, 141]. Drugs such as metformin [45], zinc [63], resveratrol [64], salidroside [78], and betulinic acid [70] may act through the AMPK/mTOR signaling pathway to promote autophagy and exert anti-inflammatory, anti-apoptotic, and antioxidant effects. Eugenol and curcumin are phenolic compounds that regulate autophagy to protect the CNS. Eugenol, one of the active components of the traditional Chinese medicine Acori Graminei Rhizoma, can alleviate brain injury by modulating the AMPK/mTOR/p70S6K signaling pathway [142]. Curcumin, extracted from turmeric, has demonstrated anticancer and antioxidant effects in clinical settings. It can promote functional recovery after SCI, reduce neuronal apoptosis, improve spinal cord integrity, and support remyelination [143].

#### Drugs that inhibit or block autophagy in SCI

3-Methyladenine (3-MA) is an effective autophagy inhibitor that suppresses the formation of autophagosomes and is widely used in research [144]. Chloroquine (CQ) disrupts the fusion process between autophagosomes and lysosomes and is another common autophagy inhibitor [145]. Both 3-MA and CQ significantly block the formation of autophagy and promote neuroprotection in SCI. Valproic acid is a clinically used anticonvulsant that significantly reduces the protein levels of Beclin-1 and LC3 following SCI [44]. Ginsenoside Rb1, one of the main active components of *Panax ginseng* (CA Meyer), has recently been shown to inhibit autophagy, reduce motor neuron loss, and promote functional recovery following traumatic SCI in rats [146]. Additionally, some agents appear to regulate autophagy in a bidirectional or context-dependent manner rather than acting as simple inducers or inhibitors. A representative example is hydrogen sulfide (H₂S) in spinal cord I/R injury. Previous studies suggest that H₂S can exert neuroprotective effects by promoting autophagy under certain pathological conditions, whereas under conditions of excessive autophagic activation it may also reduce neuronal damage by suppressing autophagic cell death. These findings indicate that the effects of H₂S on autophagy depend on the pathological context and the baseline autophagic state, and that the therapeutic goal may differ between restoring insufficient autophagy and preventing excessive autophagic injury [147, 148].

Current evidence regarding Wnt3a should be interpreted more cautiously. In SCI, the available direct evidence mainly supports that Wnt3a promotes neuroprotection, at least in part, through autophagy activation via inhibition of the mTOR signaling pathway [149, 150]. However, evidence demonstrating bidirectional regulation of autophagy by Wnt3a in SCI remains insufficient. It may therefore be more appropriate to describe Wnt3a as a context-dependent regulator of autophagy-related neuroprotection, while its broader dual effects on autophagy still require further direct validation in SCI models. Overall, these observations suggest that autophagy-targeted therapy in SCI should not be viewed as a simple “activation versus inhibition” strategy, but rather as a dynamic and context-dependent intervention.

Recent therapeutic studies further suggest that autophagy-targeted intervention in SCI is expanding beyond conventional small-molecule agents. For example, exosome-shuttled miR-5121 from A2 astrocytes was reported to promote BSCB repair by activating autophagy in vascular endothelial cells, while hADSC-based combinational therapy also improved recovery partly through autophagy modulation [151, 152].

Apart from simply promoting or inhibiting autophagy, some agents may exert bidirectional or context-dependent effects on autophagy depending on the injury model, disease stage, and baseline autophagic status. Moreover, the same agent may regulate SCI-related autophagy through different signaling pathways. In summary, targeting autophagy is a novel strategy for treating SCI. While these approaches have not yet been translated into clinical practice, they offer important insight into future therapeutic development.

The pharmacological agents reported to activate or restore autophagy/autophagic flux and those reported to inhibit or block it in SCI are summarized in Table 2 and 3, respectively.

**Table 2:** Pharmacological agents reported to activate or restore autophagy/autophagic flux in SCI

Agents	Autophagic regulation	Type of SCI model	Mechanisms and outcomes	Representative treatment timing	Action pathway	References
Rapamycin	Induction	Contusion injury Hemisection injury	Inhibits mTOR signaling, enhances autophagic flux, reduces inflammation and astrocyte proliferation, attenuates neuronal apoptosis, and promotes axonal regeneration.	Immediate (1 h post-injury), then daily for 2 days	Not reported	[58, 121, 137]
VEGF165	Induction	Contusion injury	Enhances autophagy, suppresses inflammation, improves BBB scores, and reduces motor neuron loss.	Early subacute, repeated administration	Not reported	[138]
Ezetimibe	Induction	Contusion injury	Activates autophagy and inhibits apoptosis.	Early acute (1, 24, and 72 h post-injury)	PI3K/AKT/mTOR	[139]
Melatonin	Induction	Contusion injury	Enhances autophagy, reduces apoptosis, and improves locomotor function.	Early acute, repeated administration	PI3K/AKT/mTOR, SIRT1/AMPK	[56, 140, 141]
Metformin	Induction	Compression injury; Contusion injury	Enhances autophagic flux, improves functional recovery, and suppresses apoptosis and inflammation.	Early acute, repeated administration	AMPK–mTOR–p70S6K NF-κB	[45]
Zinc	Induction	Contusion injury	Promotes neuronal autophagy and inhibits apoptosis and NLRP3 inflammasome activation.	Early acute, repeated administration	AMPK/mTOR	[63]
Resveratrol	Induction	Contusion injury	Activates autophagy, suppresses neuroinflammation and apoptosis, and confers neuroprotection.	Early acute, repeated administration	AMPK/mTOR NF-κB	[64]
Salidroside	Induction	Compression injury	Enhances autophagic flux, reduces apoptosis, and attenuates neuroinflammation.	Immediate post-injury, then daily	AMPK/mTOR	[78]
Betulinic acid	Induction	Contusion injury	Restores autophagic flux and reduces ROS accumulation.	Early acute, daily for 3 days	AMPK–mTOR–TFEB	[70]
Curcumin	Induction	Contusion injury	Reduces neuronal apoptosis, preserves spinal cord integrity, promotes functional recovery and remyelination, and suppresses inflammation.	Early acute administration	Akt/mTOR	[143]
Wnt3a	Induction	Contusion injury	Promotes autophagy and neuroprotection; context dependence remains unclear.	Early acute administration	mTOR Wnt/β-catenin	[149, 150]
H₂S	Induction	I/R injury	Induces autophagy, reduces spinal cord infarct size, and improves hindlimb motor function.	Pretreatment (I/R model)	miR-30c	[147, 148]

*SCI* spinal cord injury, *I/R* ischemia–reperfusion

**Table 3:** Pharmacological agents reported to inhibit or block autophagy/autophagic flux in SCI

Agents	Autophagic regulation	Type of SCI model	Mechanisms and outcomes	Representative treatment timing	Action pathway	References
MA	Inhibition	Hemisection injury	Blocks autophagic flux, attenuates remote degeneration, and improves spontaneous functional recovery.	Early acute administration	Not reported	[144]
CQ	Inhibition	Compression injury	Inhibits autophagy-associated inflammation and ER stress and improves locomotor function.	Early acute administration	Not reported	[145]
Valproic acid	Inhibition	Contusion injury	Reduces autophagic cell death, prevents myelin sheath damage, and promotes motor recovery.	Early acute administration	Not reported	[44]
Ginsenoside Rb1	Inhibition	Compression injury	Inhibits autophagy, reduces motor neuron loss, and promotes functional recovery.	Post-injury, repeated administration	Not reported	[146]
H₂S	Inhibition	I/R injury	Reduces oxidative stress and inhibits autophagy-associated neuronal cell death.	Pretreatment (I/R model)	AKT/mTOR	[147, 148]

**Note:** Representative treatment timing indicates the typical intervention window reported in the cited studies rather than identical dosing schedules across all experiments. *SCI* spinal cord injury, *I/R* ischemia–reperfusion

#### Phase-dependent roles of autophagy and the therapeutic window of intervention

The therapeutic value of autophagy modulation after SCI is highly phase-dependent. In the acute stage, immediate intervention may be beneficial when it restores impaired autophagic flux, promotes the clearance of damaged mitochondria and protein aggregates, and limits oxidative stress, ER stress, and early neuronal death. However, in more severe injury settings, simply increasing autophagy-related markers may be insufficient or even misleading if lysosomal dysfunction and flux blockade are not corrected. By contrast, delayed or prolonged intervention during the subacute and intermediate stages may exert greater benefit through modulation of microglial/macrophage activation, suppression of maladaptive neuroinflammation, and support of tissue remodeling and functional recovery. Therefore, the comparison between immediate and delayed administration should not be framed as a binary choice, but rather as a distinction between different pathological priorities at different stages of SCI. Therapeutic strategies that normalize autophagic flux in the acute phase and subsequently reshape the inflammatory and reparative microenvironment in later phases may offer greater translational value than uniform “activation” or “inhibition” approaches [14, 45].

### Challenges in the clinical translation of autophagy-regulating therapies

Despite the encouraging results reported in preclinical studies, the clinical translation of autophagy-regulating therapies for SCI remains challenging. First, the effects of autophagy are highly dynamic and phase-dependent, and the same intervention may be beneficial at one stage of injury but detrimental at another. Second, autophagy exhibits substantial heterogeneity across different cell types and injury models, making it difficult to define a universally effective therapeutic strategy. Third, commonly used biomarkers such as LC3-II and p62/SQSTM1 do not always reliably distinguish autophagy induction from impaired autophagic flux, especially in the presence of lysosomal dysfunction, which complicates treatment evaluation and therapeutic monitoring *in vivo*. In addition, many currently available agents regulate autophagy indirectly through upstream signaling pathways and therefore lack sufficient specificity, raising concerns regarding off-target effects and translational reproducibility. These issues indicate that future studies should focus on identifying more precise therapeutic windows, improving cell-specific targeting, and developing more reliable approaches for monitoring autophagic flux in clinically relevant settings.

## Conclusions

Secondary injury after SCI provides a modifiable therapeutic window in which autophagy may influence neuroinflammation, oxidative stress, lysosomal integrity, mitochondrial quality control, neuronal survival, and axonal repair. However, the effects of autophagy are highly context-dependent and vary with injury type, severity, stage, cell type, and baseline autophagic flux status; therefore, simply activating or inhibiting autophagy is unlikely to provide a universally effective strategy. Future studies should prioritize dynamic assessment of autophagic flux and lysosomal function, define phase- and cell-specific therapeutic windows, and develop more selective and clinically feasible modulators. Integrating these mechanistic and translational considerations may enable safer and more precise autophagy-targeted interventions and ultimately improve functional recovery after SCI.
